# How many plasmids can bacteria carry? A synthetic biology perspective

**DOI:** 10.1098/rsob.240378

**Published:** 2025-07-30

**Authors:** Cholpisit Kiattisewee

**Affiliations:** ^1^ Institute for Medical Engineering and Science, Massachusetts Institute of Technology, Cambridge, MA, USA; ^2^ Molecular Engineering and Sciences Institute, University of Washington, Seattle, WA, USA

**Keywords:** plasmids, bacteria, synthetic biology

## Abstract

Plasmids are pinnacle tools in synthetic biology and other biotechnological applications. They serve as the simplest approach to introduce recombinant DNA, which is then transcribed into RNA that functions as is or is translated into a protein of interest. Despite their widespread utility, the question ‘how many plasmids can be used in this bacterium?’ remains underexplored in the existing literature. In this article, I discuss the maintenance of multiple unique plasmids in bacteria through a microbial synthetic biology perspective, both in theoretical and practical aspects. I delve into the existing evidence of multi-plasmid systems, aiming to pinpoint the possible maximum number of unique plasmids a single microbe can carry. Finally, I highlight how the existing applications of multi-plasmid systems drive novel discovery and development in metabolic engineering, synthetic biology and other relevant areas in comparison to other non-plasmid strategies.

**Figure:**
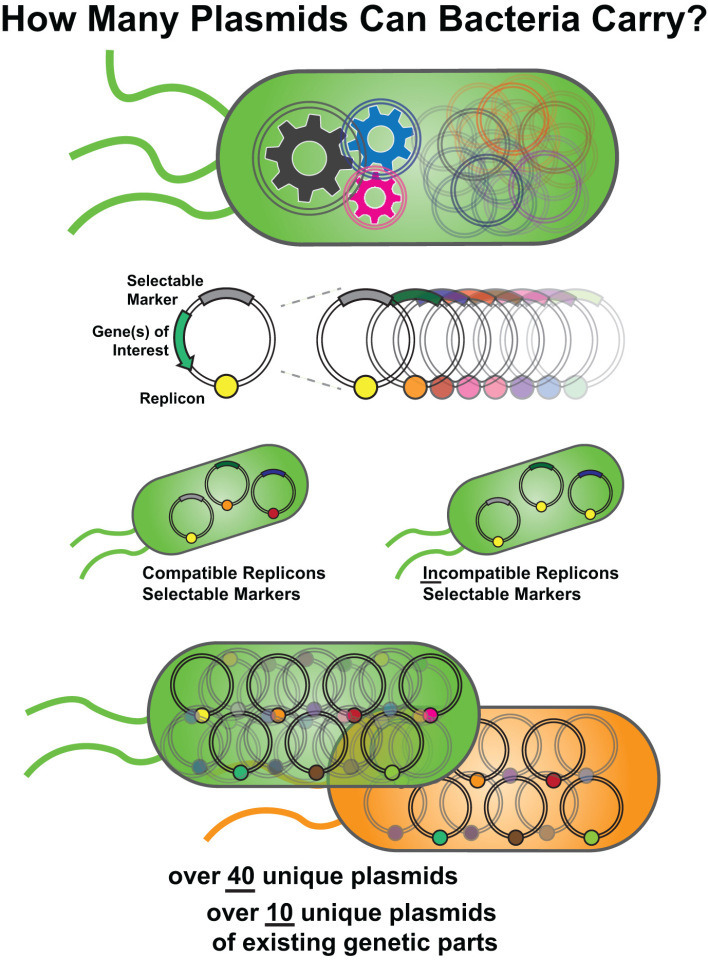

## 1. Introduction

A plasmid, a small circular DNA that replicates independently of the chromosomal DNA, is an indispensable tool in synthetic biology research. Naturally occurring plasmids usually provide increased fitness to the carrying organisms, such as metabolic capabilities, resistance to harmful chemicals or virulence factors for biological tug-of-war [1–5]. Plasmids have been repurposed in biotechnological research as vectors to deliver biological function to various hosts across kingdoms, from microbes like bacteria and fungi to higher eukaryotes or even be incorporated into engineered virus particles [6,7]. The ability to introduce foreign recombinant DNA into living organisms is the basis of genetic engineering. DNA serves as memory storage that will be transcribed into RNA messages which either function directly or are translated into proteins—key machinery of the living cell factory.

Engineering plasmids to incorporate molecular information is a fundamental task in molecular biology, alongside other DNA writing and reading techniques. Introducing a plasmid—or multiple plasmids—into a living organism alters its metabolism [8–11]. The simplest form of metabolic change is the function of biological markers used for selection of the engineered variants. In bacterial engineering, antibiotic-resistant genes (ARGs) are the most common markers as they enable bacterial growth in the presence of antibiotics while eliminating the plasmid-free variant. However, one selection marker is often paired with one plasmid species while introduction of additional plasmids requires a compatible design to achieve stable maintenance of multiple plasmids [12,13]. In spite of various guidelines surrounding plasmid compatibility [14–17], there is no direct articulation on how many plasmids can or should be introduced to a single microbe. In this perspective, I discuss how many plasmids can be carried by a single bacteria based on theoretical limits and experimental evidence in the literature. In the later part, I list examples and advantages of biological programming based on incorporation of multiple plasmids and discuss possible developments tha t arise from having a higher number of unique plasmids in a single organism. It should be noted that plasmids can also refer to linear DNA that replicate freely in bacteria, e.g. N15 phage-derived plasmid [18]. However, this article will focus on circular plasmids which are widely-used in synthetic biology applications.

## 2. Looking back at plasmids in nature

**Figure 1.**
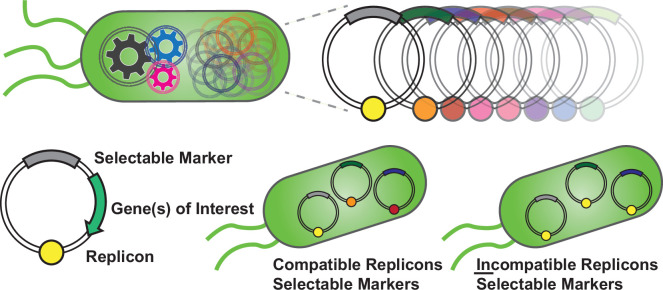
How many plasmids can bacteria carry? Multiple plasmids can replicate in bacteria when appropriate machinery is included. Important elements of a plasmid are compatible origin of replication, selection marker and arbitrary cargo of interest. Different plasmids with distinct markers and compatible replicons can be sustainably maintained over multiple generations. In the case where multi-copy incompatible replicons were used, the multi-plasmid system may be achieved but often becomes unstable over extended culture periods.

**Figure 2.**
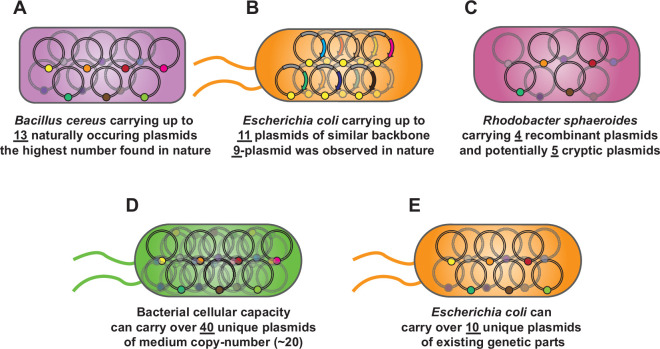
Sustainable multi-plasmid systems with compatible parts (A) up to 13 naturally occurring plasmids were found in *B. cereus*. (B) Eleven plasmids of the same replicon and markers can be simultaneously transformed in *E. coli* using a highly efficient transformation method while a nine-plasmid strain was found in the natural isolate. (C) A non-model organism, *Rhodobacter sphaeroides*, can carry four recombinant plasmids and plausibly carry nine plasmids in total, assuming that five cryptic plasmids remain intact. (D) Theoretical limits based on the reported number of plasmids suggest that bacteria can carry over 40 unique plasmids given that each replicon was engineered to be medium or low copy (<20 copy). (E) Trajectory for multi-plasmid *E. coli* with existing plasmid replicons and selection markers can be over 10 unique plasmids.

Since plasmids are naturally occurring and have been a prominent tool in bacterial metabolism, communications and evolution, the theoretical maximum number of plasmids that synthetic biologists can introduce should, in principle, surpass the natural capacity (figure 1). For *Escherichia coli*, a model organism for biological research and plasmid engineering, natural isolates have been found to carry up to nine plasmids within a single strain [19]. The highest number of unique plasmids found in a single bacteria is reported in *Bacillus cereus* with 13 plasmids records, sizes varying from 2 to 600 kb [20] (figure 2). Of note, a plasmid is usually not a necessary element and most organisms used in synthetic biology might be engineered to remove endogenous or cryptic plasmids prior to further modification.

For instance, *Pseudomonas putida* KT2440—a widely used microbe for lignin valorization—was derived from *P. putida* mt-2 by curing the pWW0 plasmid (also known as pTOL), a 117 kb plasmid responsible for degradation of toluene and xylenes [21]. Although this curing abolishes KT2440’s ability to catabolize toluene, it was necessary to avoid plasmid incompatibility issues, which will be discussed in the next section. Similarly, *E. coli* Nissle 1917—a widely used probiotic bacterium—carry two cryptic plasmids, pMUT1 (3.2 kb) and pMUT2 (5.6 kb), which can either be cured to avoid potential plasmid incompatibility [22,23] or repurposed as selection marker-free vectors [24,25]. The dispensable nature of plasmids distinguishes them from bacterial chromosomes despite both being circular DNA. Key features of bacterial chromosomes not necessarily present in plasmids include: (i) harbouring genes essential needed for growth and survival and (ii) replicating in synchronous with cell division [26], which makes them harder to insert, remove or engineer than plasmids.

To summarize from the evidence mentioned above, at least nine plasmids can be introduced into model species like *E. coli* and maybe up to 13 plasmids similar to that of *B. cereus* if there are no incompatibility problems originating from existing cryptic plasmids and engineered plasmids to be introduced.

## 3. Requirements for carriage of multiple plasmids

Plasmid maintenance is the most important factor for multi-plasmid system construction. Appropriate selection pressure is usually required when introducing two or more plasmids. When multiple plasmids are competing for cellular resources, some species of plasmid may be lost over time in the absence of appropriate selection pressure, leading to stochastic plasmids ratio. This variability is undesirable for quantitative design in synthetic biology [12,13,27,28]. To address this point, plasmids employing orthogonal replication approaches are considered compatible and preferable for building multi-plasmid systems.

Historically, each plasmid will be assigned to arbitrary ‘incompatibility groups’ (e.g. IncI for ColE1 and IncP for pBBR1), providing a guideline for molecular biologists to select plasmids from different groups to ensure maintenance in a single host [2,12]. With advancement in DNA replication knowledge, plasmid compatibility can be configured from the DNA sequence and their replication protein structure, rendering multi-plasmid programming much easier. However, it should be noted that despite having different names, some plasmids might share the same root. For example, the ColE1 plasmid family (IncI) includes pMB1, pBR322, pET, pGEM, pUC and most of the approx. 500 bp *ori* annotation present shuttle vectors. Shuttle vectors are used for molecular cloning of plasmids whose replication machinery cannot function in *E. coli,* such as origins of replication from unconventional Gram-negative bacteria, Gram-positive bacteria, yeasts or mammalian systems [10,29–31].

DNA replication of ColE1-family plasmids—thoroughly investigated in 1980s—relies on two RNA molecules: RNA-II, which serves as a primer for DNA replication, and RNA-I, an antisense RNA that regulates replication through negative feedback mechanism [32–36]. ColE1 replication also involves host machinery, DNA polymerase and ribonuclease H, resulting in a narrow host range mainly in proteobacteria [1,32,37,38]. The major difference of these ColE1-family plasmids lies in their copy numbers, which range from medium (15–20 copies for pET) to very high (up to 700 copies for pUC). This variation is due to the presence or absence of the ROP protein—which modulates RNA-I/RNA-II interactions—or some mutations that lead to significant change in expression level and interactions of these RNAs [33,39,40]. Notably, when the copy number of the plasmid is high enough, it is possible to maintain multiple plasmids of the same incompatibility group using different antibiotic selection markers [13,41]. The shared-resource trait has recently been repurposed for programmable dynamic control aided by modelling approach of systems and synthetic biology [27].

## 4. Theoretical limits based on plasmid copy number

Since plasmid copy number can vary from 1 to at least 700 copies per cell, it is possible to allocate capability to replicate those 700 DNA molecules into multiple unique plasmids at lower copy numbers. Prior to allocating copies of plasmids to unique variants, one might wonder if 700 is the maximum number of plasmids one bacterial cell can have? Rouches *et al.* had developed a ColE1-based system with tunable copy number from 1-copy to 800-copy in response to small-molecule concentration, anhydrotetracycline [42]. Additionally, Ramiro-Martínez *et al.* stated that plasmid copy numbers can exceed 1000 [43]. A model developed in this work showed that if the plasmid copy number exceeds 1000 copies, they may suffer from the runaway replication effect [44]. Although higher copy number can potentially lead to increased gene expression and later partition to larger programmes, it incurred a much higher metabolic burden to the cell [45]. The cellular resources used for plasmid replication also play a role as depicted by various studies [43,46,47]. Ramiro-Martínez *et al.* also illustrate an inverse relationship between plasmid size and plasmid copy number emphasizing the metabolic payload on extrachromosomal DNA replication.

Here, based on the evidence that 800−1000 copies of plasmid DNA can be maintained in a single bacterial cell, it is theoretically just to maintain up to 40−50 unique plasmids if all variants are 20-copy or less given that they can be properly selected and maintained. Tomoiaga *et al*. have successfully shown that 11 unique plasmids can be incorporated into a single colony of *E. coli* [48] (figure 2). However, this work did not use compatible plasmid replicons or selection markers and will not allow stable maintenance of these multiple plasmids in subsequent cultivation. To this end, I argue that the limiting factor that prevents synthetic biologists from engineering the multi-plasmid carrying microbes is not the cellular capacity of bacteria, but the finite availability of unique, compatible plasmid replicons and associated selectable markers in existing toolbox.

## 5. Repertoire of compatible multi-plasmid parts in bacteria

In the field of synthetic biology and metabolic engineering, there are various plasmid collections developed in the past decades for distinct engineering trajectories. In this article, I describe three-plasmid collections that are widely used in the field: (i) Novagen’s Duet plasmids, (ii) BglBrick platform and (iii) Standard European Vector Architecture (SEVA) collection, developed with at least four compatible plasmids in each set [49–51].

First, the Novagen Duet plasmid collection is a classical toolbox that enables simple cloning into two multiple cloning sites, the region designed for plasmid engineering, available on each Duet plasmid. Duet collection provided five compatible ColE1-like plasmids available with different antibiotic markers and copy number up for selections; (i) pETDuet−1 with Ampicillin-resistant gene (AmpR), (ii) pACYCDuet−1 with Chloramphenicol-resistant gene (CmR), (iii) pCDFDuet−1 with Streptomycin/Spectinomycin-resistant gene (SmR), (iv) pRSFDuet−1 and (v) pCOLADuet−1 where both pRSF and pCOLA carry Kanamycin-resistant gene (KmR). Designed for T7-based IPTG-inducible systems, these vectors are primarily used in protein expression and some exploratory metabolic engineering workflows [14,51].

Second, the BglBrick platform introduced a more systematic organization of plasmid components where the origin of replication and antibiotic selection markers are interchangeable due to pre-designed modular architecture [49]. The BglBrick collection provided four replicons—(i) ColE1 (same group as pET), (ii) p15A (same group as pACYC), (iii) pSC101 as a low-copy variant for *E. coli* and (iv) pBBR1 serving as a broad-host-range plasmid for application in non-*E. coli* microbes. This platform provided three widely used antibiotic-resistant markers: AmpR, CmR and KmR. BglBrick collection also provides a variety of small-molecule inducible promoters for programmable gene regulation. This ability to shuffle genetic parts opens the door to increase the number of plasmids within a single bacteria and inspires many next-generation toolsets.

Third, the SEVA collection adopted a similar modularity architecture in BglBrick with improvement for the broad-host-range aspect of plasmid toolkit. The original SEVA collection introduced five replicons—all distinct from the Duet and BglBrick collections except for pBBR1—and six antibiotic selection markers, with Gentamicin (GmR)- and Tetracycline (TcR)-resistant genes added on top of four markers in the Duet toolkit [50]. The pRK2 and pRSF1010 replicons introduced in this SEVA toolkit are known to be broadly applicable in diverse Gram-negative bacteria comparable to pBBR1. The other two replicons are narrow-range plasmids; pR6K, which require integration of *pir* gene into the host genome for replication, and pRO1600/ColE1 shuttle vector, replicative in pseudomonads. The SEVA architecture has been widely adopted globally and continuously upgraded over the years to include new replicons, selection markers and genetic cargos. Major advancements were captured in the SEVA 2.0, SEVA 3.0 and SEVA 4.0 updates, which included additional replicons of *E. coli* (p15A, pSC101, pUC and pET) [52], Gram-positive replicons [53] and compatible sister toolkits [54].

Since each collection has distinct architecture and not a single collection has encompassed all replicons curated in all three toolkits, it is possible to combine the Duet replicons with SEVA parts (pCDF, pRSF and pCOLA) together with other outstanding broad-host-range plasmids (pBAV1/pWV01, pSa, pTF2-FC2), which are potentially compatible with all other plasmids mentioned in this study [55–58]. The narrow range mini-F plasmid, originally developed from *E. coli* F plasmids, can also be incorporated into the set, yielding a total of 14 plasmids comprising five compatible ColE1-family plasmids (ColE1, p15A, pCDF, pRSF1030, pColA), four broad-host-range plasmids (pBBR1, pRK2, pRSF1010, pBAV1, pSa, pTF2-FC2) and three narrow-host-range plasmids known to be functional in *E. coli* (pSC101, pR6K, mini-F). If more compatible replicons are necessary, in the case of *E. coli*, the engineering approach to generate new orthogonal ColE1-family replicons were recently described [41,59,60].

Here, I focus on *E. coli*-compatible vectors, which are mostly inapplicable with Gram-positive bacteria unless shuttle vectors were introduced. Some modular toolkits of shuttle vectors are available for synthetic biology applications in non-model organisms [61–64]. In the case of other unconventional Gram-negative bacteria, other broad-host-range plasmids or shuttle vector systems [65–67] could be included to expand the repertoire of plasmid replicons. New compatible replicons could also be mined from metagenomic data coupled with bioinformatic strategies [1,68,69]. The full list of *E. coli*-compatible plasmid replicons mentioned in this work is listed in table 1.

**Table 1.** Plasmid replicons listed in this article.

replicon^a^	host-range	copy number in *E. coli*	size^b^
ColE1 (pMB1, pBR322, pET, pGEM, pUC)	coli and close relatives	high (very high for pUC)	~0.7 kb
p15A (pACYC)	coli and close relatives	medium	~0.7 kb
pCDF (CloDF13)	coli and close relatives	medium	~0.7 kb
pRSF1030	coli and close relatives	high	~0.7 kb
pCOLA (ColA)	coli and close relatives	medium	~0.7 kb
mini-F (F plasmid)	coli and close relatives	low	~1.4 kb (6.0 kb)^c^
pSC101	coli and close relatives	low	~1.2 kb
pR6K	only in pir + strain	medium	~0.4 kb
pBBR1	broad-host-range	medium to low	~1.4 kb
pRK2	broad-host-range	low	~2.0 kb
pRSF1010	broad-host-range	high	~5.2 kb
pBAV1 (pWV01)	broad-host-range	high	~1.5 kb
pSa	broad-host-range	low	~1.5 - 2.0 kb
pTF2-FC2	broad-host-range	low	~3.6 - 5.0 kb

^a^ Plasmid variants in the same incompatibility group are provided in parentheses.

^b^ Approximate replicon size excludes some plasmid elements such as oriT and mob genes.

^c^ The mini-F plasmid has different versions and can be as small as 1.4 kb or as large as 6.0 kb.

Regarding selectable markers, I curated antibiotic-resistant markers from the SEVA sister collections and other notable antibiotic groups to expand the repertoire. Apramycin (ApmR)-, Trimethoprim (TmpR)-, Erythromycin (ErmR)-, Zeocin (ZeoR)-, Quinolone (QnR)- and Triclosan (TclR)-resistant genes were tested to be functional in *E. coli*, providing 12 antibiotic resistance markers. Antifungal-resistant genes from yeast toolkit, NatR (Nourseothricin) and HygR (Hygromycin), are also applicable for bacterial selection and top up the markers to 14 equivalent to the number of replicons [70]. Note that NatR and HygR should be used with more precaution due to their distinct toxicity profiles to humans. The full list of *E. coli*-compatible selection markers mentioned in this work is available in table 2.

**Table 2.** Plasmid selection markers mentioned in this article.

marker	selection	function
AmpR	ampicillin or carbenicillin	β-lactamase degrades ampicillin or other β-lactam
CmR	chloramphenicol	chloramphenicol acetylation
KmR	kanamycin	aminoglycoside phosphorylation, acetylation, or adenylation
SmR	streptomycin or spectinomycin	aminoglycoside phosphorylation or adenylation
GmR	gentamicin	aminoglycoside acetylation or adenylation
ApmR	apramycin and gentamicin	aminoglycoside acetylation or adenylation
TcR	tetracycline	efflux pumps
TpmR	trimethoprim	dihydrofolic acid reductase that trimethoprim cannot bind
ErmR	erythromycin	rRNA modification by methyltransferase
ZeoR	Zeocin	resistant protein interferes with zeocin binding to DNA
QnR	nalidixic acid	protect DNA gyrase
TclR	triclosan	alter cell membrane structure
NatR	nourseothricin	aminoglycoside acetylation
HygR	hygromycin	aminoglycoside phosphorylation
*ΔdapA*	2,6-diaminopimelic acid auxotroph	inability to grow without this gene
*ΔthyA*	thymidine auxotroph	inability to grow without this gene
*ΔglmS*	N-acetyl-D-glucosamine auxotroph	inability to grow without this gene
sacB	counterselection of sucrose	turns sucrose into toxic compound

Selection markers on this table were listed with generalization of gene names. The actual gene name might be referred to differently depending on their functions. For instance, kanamycin resistant genes in literature may refer to phosphorylation, acetylation, or adenylation enzymes. Some antibiotic resistant markers can also confer resistance to multiple antibiotics, e.g. ApmR from SEVA toolkit yields resistance to both apramycin and gentamicin.

In this context, it should be noted that pR6K requires a specific strain of *E. coli* with *pir+* genotype to enable its replication and that Apramycin-resistant gene confers promiscuous activity to gentamicin [71].

## 6. Construction of a multi-plasmid carrying bacteria

Based on the genetic parts repertoire described in the previous section, it is now feasible to surpass the 13-plasmid system found in nature*,* especially with emerging synthetic elements. In my experience, five plasmids of the ColE1-family carrying their corresponding Duet marker pair, with pRSF1030 carrying GmR instead of KmR, can be transformed into a single *E. coli* cell. Recent work from Liu *et al.* found that co-transforming ColE1-replicon plasmids with six distinct selection markers (AmpR, CmR, KmR, SmR, ApmR and TcR) yielded up to four-plasmid systems. However, by engineering the replicons into orthogonal SynORI systems, six-plasmid bacteria can be achieved and remain stable for a week with maintained selection pressure [41]. Nonetheless, this attempt to answer the basic question about the number of plasmids also led to the introduction of a multi-drug resistance trait, a global threat to mankind. Therefore, I suggest that we refrain from introducing more plasmids with antimicrobial-resistant genes (ARGs) as selection markers and explore other options that do not involve generation of multi-drug-resistant organisms.

Recently, Amrofell *et al.* have constructed an ARG-free selection marker for plasmid cloning and maintenance using auxotrophic markers and select on engineered *E. coli* strains [72]. Including this work, at least three auxotrophic markers can be used in rich media, e.g. Lysogeny Broth (LB): (i) 2,6-diaminopimelic acid (DAP) for *ΔdapA* [73,74], (ii) thymidine (Thy) for *ΔthyA* and (iii) N-acetyl-D-glucosamine (NAcG) for *ΔglmS* [72]. For selection in minimal media, a pool of auxotrophic markers—such as amino acid requirements—are described in the KEIO *E. coli* knock-out mutant collection [75]. Beyond antibiotic and auxotrophic markers, plasmid selection can also repurpose toxin–antitoxin systems [76] or counterselectable markers. For example, the Nanoplasmid vector [77] uses antisense RNA to represse *sacB* gene, enabling sucrose-based selection. Despite not having tested the hypothesis on the maximum number of plasmid yet, I propose that this question be experimentally tested in a safer route utilizing safe markers on an engineered strain with a biocontainment circuit installed, if applicable.

Finally, I want to emphasize that this proposed construction of over 10 unique plasmids can be achieved only in model organisms like *E. coli* where a repertoire of plasmids is much more diverse than non-model microbes beyond Enterobacteriaceae. In my personal experience working with non-*E. coli* systems, I found that broad-host-range plasmids tend to be more burdensome than ColE1-family systems in *E. coli* due to (i) their relatively larger replicon size (1.5−5.0 kb for broad-host-range compared to approx. 700 bp of ColE1-family) and (ii) the requirement to fully express replication proteins while the ColE1-family relies on RNA-I/RNA-II machinery independent of plasmid-borne protein [2,3]. Utilizing a two-plasmid system in *P. putida* KT2440 based on pBBR1 and pRK2 introduces significant metabolic burden [78,79]. Introducing multiple unique plasmids into *Bacillus subtilis* strain 168 and *Acinetobacter baylyi* ADP1 is possible but remains challenging. Despite successful introduction of two plasmids into *B. subtilis*, transformation of the second plasmid species into a plasmid-containing *B. subtilis* yielded much less transformation efficiency, suggesting accumulated metabolic burden from an increasing number of unique plasmids. In *A. baylyi*, I observed recombination between two plasmid species occasionally. This might be due to strong capability of *Acinetobacter* to take up DNA and recombine, which make multi-plasmid maintenance highly challenging. Similar effects were also reported for *B. subtilis* and highly recombinant phagemid systems [13,80]. These challenges on available replicons, metabolic burdens and recombination issues remain concerning for engineering campaigns of unconventional microbes emerging in the synthetic biology research landscape.

Although not obviously depicted, there are various reports on multi-plasmid systems in non-model microbes. Multiple broad-host-range plasmids like pBBR1 derivatives can be efficiently introduced and maintained in methane-utilizing bacteria *Methylomicrobium extorquens* AM1 given that their antibiotic-resistant markers were different [81]. This report is equivalent to the previous observation in *E. coli* for ColE1-family variants [13,27]. Similarly, the *M. extorquens* strain carrying more unique plasmids exhibited a lower growth rate as a result of increased metabolic payload. I also observed the same phenomenon in *A. baylyi* using two pRSF1010 plasmids bearing distinct ARGs. *Rhodopseudomonas palustris*, a CO_2_-utilizing photosynthetic microbe, has only a handful of compatible plasmids and requires both a broad-host-range plasmid (pBBR1) and an *E. coli-R. palustris* shuttle vector (pMG101) for a two-plasmid strain engineering system [82].

Interestingly, multi-plasmid systems in *Rhodobacter sphaeroides* were reported to carry two (pIND and pRK2) to four unique plasmids simultaneously (adding pBBR1 and pLV106) [83,84] in addition to five cryptic plasmids assumed to remain intact [85] (figure 2). Additionally, the ColE1-family plasmids might be applicable in some proteobacteria with emerging interests. Established toolkits for fast-growing *Vibrio natriegens* [86] and microbial fuel cell *Shewanella oneidensis* [87] suggested that ColE1 and p15A vectors can successfully replicate in these two emerging microbes. Additionally, the ColE1 plasmid can be transformed into *A. baylyi* as suggested by a recent report on automated molecular cloning [88]. SynORI replicons, based on engineered ColE1 replicons, are also functional in *S. oneidensis* [41]. Therefore, co-maintenance of ColE1, p15A and other ColE1-family plasmids, theoretically, should also be achievable in these non-*E. coli* bacteria.

## 7. Advancement in synthetic biology enabled by multi-plasmid systems

Deploying multiple recombinant plasmid species in a single organism often imposes metabolic burden to the microbe, yet it remains attractive in synthetic biology platforms. Compared to the construction of one large plasmid or chromosomal integration, the multi-plasmid approach allows parallelized engineering of different plasmid modules. This modularity facilitates both building and testing of the Design-Build-Test-Learn processes. Chromosomal integration typically involves multiple steps: genome insertion, marker recycling and plasmid curing.

For the single-plasmid strategy, combinatorial profilings of molecular programs requires extensive molecular cloning efforts for each condition. Building large genetic programs into a large plasmid (>20 kb) also significantly impairs the DNA transformation efficiency. In this section, I present case studies that demonstrate how multi-plasmid systems can accelerate synthetic biology discovery with advantages over genome integration of single-plasmid approaches.

### 7.1. System metabolic engineering with a multi-plasmid system

**Figure 3.**
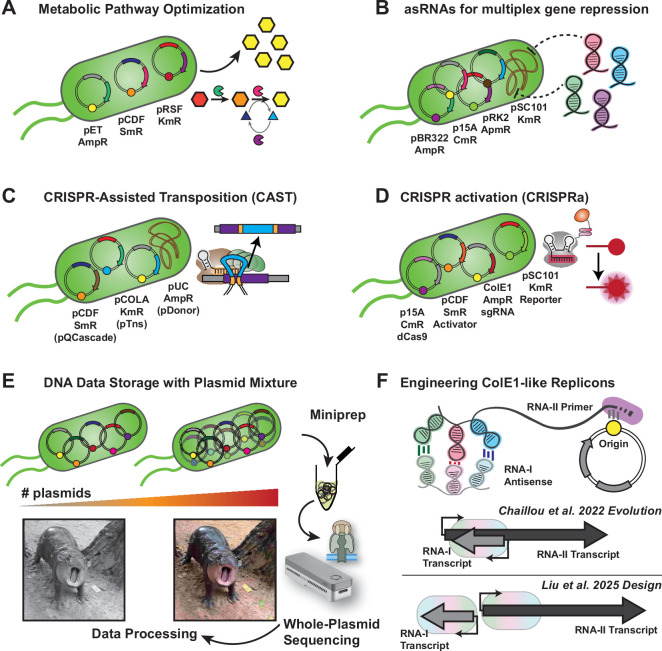
Synthetic biology applications of multi-plasmid systems. (A) Multi-plasmid systems for metabolic pathway engineering. Different metabolic modules were allocated to each plasmid and combinatorial profiling can be achieved by mixing variants of each module. (B) Utilization of compatible plasmids expressing different paired termini antisense RNAs (PTasRNAs) each targeting endogenous genes of bacteria. (C) The development of CRISPR-assisted transposition (CAST) system segregates each module on separate plasmids. Once identifying optimal conditions, combining all modules into a single plasmid drastically improved its efficiency. (D) Screening of effective CRISPRa by dissecting each part into four different plasmids. Cas protein, effector, sgRNAs and target DNA can be selected and engineered orthogonally, thus accelerating synthetic biology exploration. (E) A low- and high-quality image of Moo Deng, an iconic pygmy hippo from Thailand, represents data stored in multi-plasmid systems of different numbers of unique plasmids. (F) Engineering strategies of ColE1-like plasmid to generate novel compatible replicons with two examples listed. Chaillou *et al.* [59] engineered the shared element of primer and antisense RNA while Liu *et al.* [41] segregate two parts for orthogonal engineering interactive element and concentration.

The first and simplest example is the systems metabolic engineering work by Jaroensuk *et al.* [14]. In this work, different modules for chemical production and cofactor regenerations were constructed on different plasmid systems to enable sufficient alkane production (figure 3A). Core metabolic pathways of medium-chain alkane synthesis were constructed using Duet plasmid collections (pET and pCDF). Rooting from biochemical characterization, they posited that NADPH regeneration and hydrogen peroxide degradation were the limiting factors for alkane production. Here, NADPH-regenerating enzyme and/or peroxide degradation partners were introduced on the additional pRSFDuet−1 backbone. By sequential plasmid incorporation, they found that NADPH regeneration exhibited the largest improvement compared to other potentially useful modules. This work emphasizes an advantage of pathway modules division onto multiple plasmids to minimize the molecular cloning tasks. A three-plasmid pathway using the BglBrick platform was also described by the Lee laboratory, the original inventor of the BglBrick collection [89].

### 7.2. Multi-gene regulations with regulatory RNAs on different plasmids

In gene regulation technologies, multiple plasmids can carry unique regulatory parts for perturbations of different gene targets. For example, programmable gene regulation based on antisense RNAs (asRNAs) and CRISPR guide RNAs (gRNAs) can be individually installed on each plasmid. Nakashima *et al.* developed a paired termini antisense RNAs (PTasRNAs) system, which is small and can be deployed on any plasmid of choice [16]. They tested multi-gene repression by introducing four different PTasRNAs on each compatible plasmid: p15A, ColE1, pSC101 and pRK2 (figure 3B). With each plasmid targeting a single gene, multi-gene repression programs can be readily achieved through the co-transformation of selected plasmid sets. This strategy also extends to CRISPR-based gene regulation, enabling multi-layer gRNA circuits using different plasmid types, where differences in copy number—ColE1 (high) or pCDF (medium)—offer an additional layer of control to the dynamics of genetic perturbation [90,91].

### 7.3. Optimization of synthetic biology tool with multi-plasmid system

Multi-plasmid systems have been a hallmark approach for tool building in synthetic biology. The ability to combinatorially mix different parts installed on orthogonal plasmids facilitates the workflow from both genetic part building and multi-part testing aspects. Evident examples are the novel CRISPR technology where optimization of different parts and their corresponding expression levels are usually necessary.

CRISPR-assisted transposase (CAST) has become a game-changing tool in organism and microbiome engineering in the past years [92]. In the original development and optimization of this technology, three compatible plasmids were used to identify the best combination of different parts; (i) QCascade CRISPR machinery on pCDF-SmR, (ii) transposase effector parts on pCOLA-KmR and (iii) transposon DNA donor cargo on pUC-AmpR (figure 3C). Klompe *et al*. investigated each module through one-at-a-time changes and explored the best combination of this CAST system. Later, the team took another step to combine the CRISPR and transposase machineries into a single plasmid, pEffector on pCDF-SmR, which enables cross-validation between two orthogonal CAST systems to their cognate pDonor plasmid [93]. Further engineering to enable CAST in non-model microbes and microbiome engineering was achieved by pooling all required machinery (pEffector and pDonor) into a single broad-host-range plasmid, pBBR1, which significantly enhanced transposition efficiency. This process emphasizes the power of multi-plasmid systems as a strategy to explore combinatorial space of new technology which could later be combined into a compact system afterwards for improved efficiency.

Another CRISPR technology that is difficult to engineer is the CRISPR-based gene activation. Originally, nuclease deactivated Cas9 protein (dCas9) has been repurposed for transcriptional repression, termed CRISPR interference, by blocking the initiation or elongation processes of RNA polymerase (RNAP)—using dCas9 plasmid on p15A-CmR and variation of single guide RNAs (sgRNAs) on ColE1-AmpR [94]. However, the gene activation counterpart, coined as CRISPR activation (CRISPRa), requires an additional effector for RNAP recruitment function which can be directly fused to dCas9 [95] or tethered by RNA–protein or protein–protein interactions [17,96], or deployed in multiplex manner [97]. The target of CRISPRa also plays an important role in effective gene activation and is usually deployed on a low-copy pSC101 plasmid [90,98]. Therefore, the machinery needed to optimize CRISPRa systems are (i) dCas9 or other dCas proteins [99], (ii) sgRNAs or their engineered variants, (iii) activator proteins with recruitment moiety and (iv) reporter genes with systematically programmed promoter region. To this extent, Villegas-Kcam *et al.* have modularly optimized bacterial CRISPRa using (i) circularly permuted dCas9 variants fused with SYNZIP domain on p15A-CmR, (ii) multiple RpoA-subunit from diverse organisms—also fused with a cognate SYNZIP domain for dCas9 attachment—on pCDF-SmR, (iii) collections of sgRNAs expressed on ColE1-AmpR and (iv) systematically tiled reporters pSC101-KmR (figure 3D). This four-plasmid system is one of a few examples in the field of synthetic biology that clearly demonstrate the advantage of multi-plasmid deployment with up to four modular parts on their designated vectors in a single microbe.

Notably, once achieving optimal constructs, CRISPR machinery can be consolidated into a lower number of plasmids or even a single plasmid if some parts were integrated onto the genome [15,78,98, 97]. Decreasing the number of plasmids usually increases the effectiveness of CRISPRa plausibly by mitigating plasmid replication burden and can be executed after the design rules were characterized with multi-plasmid systems. This improvement might be a result of the ‘hook effect’ mitigation which arises when concentration of one component is too high in multi-part biochemical complexes [100,101]. Therefore, plasmid copy number should be chosen appropriately depending on their biochemical roles—for instance, dCas9, which needs to be assembled with all accessory modules, is best expressed from medium- to low-copy plasmids.

Again, the multi-plasmid systems described here are mostly in *E. coli* due to a smaller pool of compatible plasmids in non-model bacteria. Lack of multi-plasmid systems in some non-model organisms is a major factor hampering the pace of technological advancement. Therefore, tool building and characterization of novel hosts remain core foundational research for microbial synthetic biology [55,78,102–104]. Nevertheless, despite limiting plasmid repertoire in *R. sphaeroides*, Tong *et al*. constructed a four-plasmid system based on pIND, pRK2, pBBR1 and pLV106 vectors to optimize the production of long-chain-free fatty acids, demonstrating the challenging multi-plasmid system in non-model microbial engineering [84] (figure 2).

### 7.4. Potential of multi-plasmid system in DNA data storage

Building on this article’s narrative to introduce multiple unique plasmids into a single microbe, it is also possible to apply the multi-plasmid system to DNA data storage and retrieval technology. While information technologies are mainly based on digital 2-bit systems, DNA data storage offers unique advantages for some specific applications [105]. DNA is incredibly dense, with a four-digit system, and stable under extreme conditions—making it a promising medium for long-term data storage for extreme cases like space missions [106]. In this context, multi-plasmid systems represent one practical strategy for encoding, amplifying and retrieving information within living cells. While DNA can be chemically synthesized in linear format, a circular plasmid tends to be more resistant to enzymatic degradation [107]. DNA in living organisms is mostly in a circular form and other components inside the cells could significantly enhance DNA durability [108]. In the literature, DNA can be stored by (i) integrating into the bacterial genome(s) with CRISPR technology [109,110], (ii) assembled as large artificial chromosomes [111] or (iii) as a mixture of high-copy plasmids in bacterial consortia [112].

Storing data in microbial DNA provides another advantage due to an ease of data propagation—equivalent to copy and paste—simply by seeding new cultures or rejuvenating the frozen or freeze-dried stocks. *E. coli* bearing multiple plasmids for long-term storage of DNA offer a cheap multiplex data retrieval option using whole-plasmid nanopore sequencing (figure 3E), by focusing on relatively small plasmids (<25 kb) appropriate for routine whole-plasmid sequencing services of miniprep DNA, e.g. Plasmidsaurus platform [113,114]. The increasing number of plasmids act as a proxy to the memory volume that a single microbial cell can carry. Nevertheless, sequencing depth of each unique plasmid is highly dependent on plasmid copy number. For instance, when a miniprep of *E. coli* bearing three unique plasmids of different copy number—ColE1 (high), p15A (medium), and pSC101 (low)—were analysed, the high abundance of ColE1 usually dominates the pooled sequencing and limits the efficiency of pSC101 sequencing. Thus, fine-tuning of plasmid copy number might be necessary to optimize the data retrieval process of multi-plasmid DNA data storage.

Engineering at replication machinery [33,115,116] or introducing replication control circuits [42,117,118] were established in *E. coli* and other microbes. When multiple ColE1-like plasmid were present, the copy number of each plasmid might also change as they share similarity in replication machinery [34,59,119]. Chaillou *et al.* and Liu *et al.* [41,59] developed an engineering strategy to develop novel compatible ColE1-like plasmids beyond replicons listed in this article (figure 3F). OriGen, a generative AI-based strategy was also recently developed, potentially providing an alternative design workflow for novel replicon construction [60]. These recent developments underscore the growing interest of multi-plasmid systems in synthetic biology. Since the replicon size, approx. 700 bp of ColE1-family is much preferred in multi-plasmid data storage over plasmid that require incorporation of replication protein (>1.5 kb) to attribute the majority of plasmid DNA for encrypted data. Other small replicon candidates include pICOt2 (approx. 900 bp) and Nanoplasmid (approx. 500 bp) systems [77,120]. With methods to construct new replicons and fine-tune their copy number, at least 10 or even over 40 orthogonal small ColE1-like replicons with comparable copy numbers should be achievable (figure 2). Recent synthetic biology advancements, such as incorporation of non-canonical nucleic acids and diverse DNA methylation patterns [121–123], can also be incorporated to expand the capacity of this multi-plasmid system. Altogether, these case studies demonstrated the benefit of multi-plasmid systems in bacteria and underscore their potentials in synthetic biology tool development.

## 8. Comparison of multiple small plasmids to a single large chromosome

While this narrative highlights the use of multiple small plasmid (<25 kb) systems for their unique advantages in synthetic biology, it is also worth comparing the biological property and distinct application that a single large synthetic chromosome might offer. To begin with, some naturally occurring plasmids—known as megaplasmids—can be bigger than some bacterial chromosomes [124]. Megaplasmids can exceed 350 kb—roughly 10% of median bacterial genome size—with the record of 2.5 Mb [125]. On the other hand, *Nasuia deltocephalinicola*, a bacterial symbiont in leafhoppers [126], has the smallest reported chromosome with only 112 kb, smaller than the well-studied pWW0 (117 kb) from *P. putida* mt-2.

Additionally, the rule that bacteria should possess only a single chromosome has been negated by *Vibrio cholerae* and its relatives, which possess two unequal-sized chromosomes [127]. It was hypothesized that the secondary plasmid evolved from fusion of multiple plasmids into megaplasmids, which later acquired the essential genes and partitioning elements of chromosomal DNA [124]. It is possible to introduce the second chromosome into bacteria or collapse two chromosomal DNAs into one [128,129]. This blurred distinction between plasmids and chromosomal engineering aligns closely with the field synthetic genomics, where the entire genome can be rewritten and reintroduced to create synthetic life forms that are unlikely to occur naturally. The clearest example is culturable *Mycoplasma mycoides*, with a 1.2 Mb chromosome, which was engineered into a minimal synthetic genome variant, JCVI-syn3.0, having only 531 kb as its chromosome [130].

To create bacteria with a synthetic genome, the bacterial genome was re-designed and assembled as a plasmid-like particle in its intermediate assembly host—in the form of a yeast artificial chromosome (YAC). Upon transplantation into the bacteria and subsequent ‘booting-up’ [131], the YAC is then considered a true chromosome in the synthetic cells because synthetic bacteria cannot proliferate without this YAC. Even though the creation of synthetic bacterial cells have improved over the past decades, it remains challenging for synthetic biology teams with lower resources to conduct whole genome synthesis and following installation. While multiplex engineering using recombination or transposition is attractive for engineering single-copy core metabolic genes, recent high-throughput methods typically limited to a few simultaneous edits and often rely on pre-installation of landing sites [132,133]. Therefore, the multi-plasmid strategy is more suitable for combinatorial engineering which remains operational with basic molecular biology and microbiology instruments.

Another intriguing question that arises at the intersection of multi-plasmid systems and synthetic genomics is whether it is possible to engineer a microbe composed of only multiple smaller plasmids—functionally analogous to how eukaryotes segregate their DNA across multiple chromosomes. In bacteria, the highest number of chromosomes reported is two, while in archaea, the classification remains controversial whether the some circular DNA molecules are true chromosomes or megaplasmids [134]. Since a minimal set of genes for creating a synthetic genome is only 531 kb, it is theoretically possible to segment the genome into multiple smaller plasmids (<100 kb) [130]. Note that the plasmid copy number will approach low- to single-copy at increasing plasmid size and at the same time elevate the difficulty to transform [43]. This point summarizes a clear distinction of plasmid and chromosome and highlights the rapid-prototyping feature offered by the multi-plasmid approach.

## 9. Conclusion

This open question article discusses the inquiry surrounding plasmid engineering and maintenance. I reviewed the theoretical limit and experimental evidence of ‘how many unique plasmids a single bacteria can possibly carry?’. By delving into the natural systems and existing synthetic biology toolkits, I propose that more than 10 unique plasmids can be readily introduced into model bacteria with existing replicons and selection markers. Upon novel design and characterization of new parts with appropriate copy-number control, installing over 40 plasmids into a single bacteria should be feasible. The bottleneck lies within the limited compatible parts, both replicons and selectable markers. The applications of multi-plasmid systems in the field of synthetic biology were described particularly when at least three or more plasmids were used in a single microbe. These multi-plasmid systems exhibited engineering potential for combinatorial investigation of new metabolic pathways, multi-gene regulations, optimization of state-of-the-art CRISPR technologies, and the applicability in the area of DNA data storage and synthetic genomics. Ultimately, while maintaining three or more plasmids is easily accessible in *E. coli*, it remains a challenge in non-model bacteria. Key barriers to introducing multiple plasmids include limited availability of replicons, potentially high metabolic burden imposed by broad-host-range vectors, mainly from the necessity to express high amount proteins as selection markers and cryptic elements and the inherent recombination activity of non-model microbes. These challenges underscore the need for continued developments of novel synthetic biology toolkits tailored towards multi-plasmid systems creation.

## Data Availability

This article has no additional data.
